# Changes in Host Immune–Endocrine Relationships during Tuberculosis Treatment in Patients with Cured and Failed Treatment Outcomes

**DOI:** 10.3389/fimmu.2017.00690

**Published:** 2017-06-15

**Authors:** Léanie Kleynhans, Sheena Ruzive, Lizaan Ehlers, Lani Thiart, Novel N. Chegou, Magda Conradie, Magdalena Kriel, Kim Stanley, Gian D. van der Spuy, Martin Kidd, Paul D. van Helden, Gerhard Walzl, Katharina Ronacher

**Affiliations:** ^1^SA MRC Centre for TB Research, DST/NRF Centre of Excellence for Biomedical Tuberculosis Research, Division of Molecular Biology and Human Genetics, Faculty of Medicine and Health Sciences, Department of Biomedical Sciences, Stellenbosch University, Cape Town, South Africa; ^2^Division of Endocrinology and Metabolism, Faculty of Medicine and Health Sciences, Department of Medicine, Stellenbosch University, Cape Town, South Africa; ^3^Centre for Statistical Consultation, Stellenbosch University, Stellenbosch, South Africa; ^4^Translational Research Institute, Mater Research Institute, The University of Queensland, Brisbane, QLD, Australia

**Keywords:** tuberculosis, biomarkers, immune, endocrine, cytokines, metabolic hormones, steroid hormones

## Abstract

A bidirectional communication between the immune and endocrine systems exists and facilitates optimum responses in the host during infections. This is in part achieved through changes in secretion patterns of hypothalamic hormones induced by inflammatory cytokines. The aim of this study was to elucidate the immune–endocrine alterations during tuberculosis (TB) treatment in patients with cured and failed TB treatment outcomes. Blood samples were collected from 27 cured and 10 failed patients and hormone as well as cytokine concentrations quantified at baseline, week 4, and month 6 of TB treatment. Hormone profiles of the two treatment outcome groups were different from each other prior to as well as during TB treatment. Treatment response effects were observed for cortisol, estradiol, T3, T4 ghrelin, leptin, amylin, adiponectin, and dehydroepiandrosterone (DHEA). Trends suggest that T4, amylin, and DHEA concentrations were different between treatment outcomes, although these did not reach statistical significance. Relationships between endocrine and inflammatory markers and the biological pathways involved differed between cured and failed treatment patients. These results highlight the complex interaction between the endocrine and immune system during active TB disease and throughout treatment and suggest that endocrine markers in conjunction with inflammatory markers may be useful in predicting unfavorable treatment outcomes.

## Introduction

Tuberculosis (TB), caused by *Mycobacterium tuberculosis* (*M.tb*), is a major global health threat, particularly in developing countries and continues to claim approximately two million lives every year (1). According to the World Health Organization, South Africa is estimated to have the highest TB incidence at 860 per 100,000 population (1). The estimated detection rate for TB increased from 58 to 69% between 2000 and 2013 (1). However, approaches to control TB have failed to contain the epidemic. In addition, rapidly increasing rates of recurrent TB episodes and increasing rates of multi-drug resistant (MDR) TB present a startling public health emergency (2).

In response to an infection, cytokines like interleukin (IL)-1, IL-6, and tumor necrosis factor (TNF)α, produced by immune cells, can activate the hypothalamus–pituitary–adrenal (HPA) axis, which results in the secretion of cortisol (3). These cytokines furthermore affect the functioning of the hypothalamus–pituitary–thyroid (HPT) axis (4) as well as the hypothalamus–pituitary–gonadal (HPG) axis (resulting in decreased secretion of androgenic hormones) (5, 6). Cortisol, in turn, inhibits the mycobacterial antigen-induced cytokine response of peripheral blood mononuclear cells (7, 8). This communication pattern exists due to the fact that cytokine-producing cells as well as hormone-producing cells share common receptors and ligands (9). This bidirectional communication between the immune and endocrine system facilitates optimal responses and maintains homeostasis in the host.

Immune and endocrine alterations were observed in TB patients, characterized by increased cortisol, estradiol, prolactin, growth hormone, thyroid hormone, and dopamine concentrations, which were accompanied by increased interferon (IFN)γ, TNFα, C-reactive protein (CRP), IL-1α, IL-6, and IL-10 concentrations when compared to healthy controls (4, 10–12). TB patients furthermore had decreased dehydroepiandrosterone (DHEA) and testosterone concentrations, alterations that were more evident in individuals diagnosed with advanced TB disease. Leptin concentrations were decreased and ghrelin concentrations increased with increasing disease severity (11, 13, 14). Epinephrine concentrations were also lower in TB patients (12). Differences in hormone concentrations in the context of TB were mostly evaluated at baseline with limited reference being made to endocrine profile changes during TB treatment. In one such study, Bongiovanni et al. showed that cortisol plasma concentrations decreased during TB treatment together with IL-6, CRP, and IL-1β (15). Additionally, DHEA plasma concentrations increased during TB treatment.

The aim of this study was to elucidate the immune–endocrine alterations during TB treatment in a cohort of HIV negative TB patients with different treatment outcomes. The study collectively investigated the concentrations of 12 hormones, ranging from peptide to steroid hormones, during the course of TB treatment in cured patients and in patients with treatment failure. These included cortisol, DHEA, T3, T4, adiponectin, leptin, ghrelin, active amylin, total amylin, growth hormone, estradiol, and progesterone. Furthermore, the association of the hormone concentrations with 30 immune markers including cytokines, chemokines, growth factors, soluble receptors, acute phase proteins, and matrix metalloproteinases (MMPs) was determined. This study is unique in that it is the first treatment response study that investigates alterations in hormone concentrations in patients with two different treatment outcomes, namely cured and treatment failure. We show that the hormone profiles change during TB treatment and that the concentrations of different hormones correlate with the concentrations of cytokines. These results emphasize the complex interaction between the endocrine and the immune systems during TB treatment and suggest that hormone concentrations in conjunction with inflammatory marker concentrations may be useful as biomarkers for TB treatment response.

## Materials and Methods

### Ethics Statement

Ethical approval was obtained from the Health Research Ethics Committee of the University of Stellenbosch and the City of Cape Town City Health. The study was conducted according to the Helsinki Declaration and International Conference of Harmonization guidelines. Written informed consent was obtained from all study participants.

### Study Subjects

Study participants, 27 cured and 10 failed TB patients, were enrolled and treated at 5 TB clinics surrounding Tygerberg Hospital in Cape Town, South Africa, between May 1999 and July 2002, as part of the Action TB study (16). The HIV uninfected pulmonary TB patients were all from the South African Colored ethnic group and were untreated at the time of enrollment with a first episode of TB. All TB patients received directly observed treatment, which consisted of an intensive phase (2 months) of rifampicin (RIF), isoniazid (INH), pyrazinamide, and ethambutol (EMB) followed by a continuation phase (4 months) of RIF and INH. The 2-month intensive phase was prolonged to 3 months if smear conversion did not occur at 2 months. The percentage of individuals requiring extended intensive phase was 15 and 45% for the cured and failed group, respectively. Patients were between the ages of 20 and 65 years. Patients were matched based on their gender and sample storage time. Individuals who had two consecutive negative culture results at the end of treatment were regarded as successfully cured whereas those who had a positive culture result at month 6 had failed treatment. All individuals adhered to treatment (>80% of drugs taken) and were infected with drug sensitive *M.tb* strains. No distinct differences in strain types were observed between failed and cured patients. Patients were excluded if they previously had TB; MDR TB, were HIV positive, presented with diabetes, malignancy, lung cancer, chronic bronchitis, or sarcoidosis, were on steroid treatment or were pregnant. Chest X-rays (CXRs) were only obtained at baseline and were read independently in a blinded fashion by a pulmonologist or clinician as previously described (17).

### Sample Collection and Processing

Samples were collected between 09h00 and 12h00 every day. Clinical information on age, sex, weight, and height (BMI) was recorded. Sputum samples were obtained at baseline and at week 4 and month 6 of treatment. Sputum smear microscopy was done using the Ziehl–Neelsen method and quantitative smear grading done according to the International Union Against Tuberculosis and Lung Disease guidelines (18). The same sample that was used for sputum smear microscopy was used to determine time-to-positivity in liquid culture using the BACTEC 12B liquid radiometric method (BD, NJ, USA) as previously described (16). At each of the indicated time points, blood was drawn, and the serum and plasma aliquoted and stored at −80°C.

### Quantification of Serum Cytokine and Plasma Hormone Concentrations by Multiplex Bead Array

Thirty cytokines, chemokines, growth factors, soluble receptors, and acute phase proteins were measured in serum using different Linco-plex kits (Millipore, Billerica, MA, USA). These were regular sensitivity kits for Eotaxin, G-CSF, GM-CSF, GRO, IFNα2, IFNγ, IL-1α, IL-1β, IL-9, IL-12(p40), IP-10, MIP-1α, MIP-1β, TNFα, TNFβ, and VEGF; high sensitivity kits for IL-5, IL-8, IL-10, IL-12(p70), and IL-13; soluble receptor kits for sIL-2Rα, sIL-4R, sIL-6R, and sVEGFRI; acute phase protein kits for CRP, SAP A, and SAP P; and MMP kits for MMP-2 and MMP-9. Metabolic hormone kits (Merck-Millipore, MI, USA) were used to quantify plasma concentrations of amylin (active), leptin, and ghrelin. T3, T4, cortisol, estradiol, and progesterone concentrations were quantified using steroid/thyroid hormone kits (Merck-Millipore). Assays were done according to the manufacturer’s instructions. Concentrations of the markers were measured on a Bio Plex platform (Bio-Plex™, Bio-Rad Laboratories, Hercules, CA, USA). All samples, quality controls (QCs), and inter-plate control were run in duplicate. Concentrations of all the analytes in the QCs were within the expected ranges and the inter-plate variation below 20%. The data generated were managed using Bio-Plex Manager Software, version 4.1.1.

### Quantification of Plasma Hormone Concentrations by ELISA

Plasma concentrations of DHEA (DRG Instruments GmbH, Marburg, Germany), growth hormone (Abnova, Taipei City, Taiwan), adiponectin (Merck-Millipore), and total amylin (Merck-Millipore) were measured by ELISA according to the manufacturer’s instructions. Hormone concentrations were detected by a Bio-Rad Microplate reader with Microplate Manager Software, version 5.2 build 103 (Bio Rad Laboratories).

### Data and Statistical Analysis

For multiplex assays, data were analyzed using the R programming statistical package (The R Foundation for Statistical Computing, Vienna, Austria). Missing values were imputed using random-forest imputation from the mice package. Data for two participants could not be imputed and were excluded from the analysis on hormone data. Fifty imputed data sets were generated for analysis. Values that were too low to be extrapolated by the Bio-Plex Manager Software were randomly imputed in each of the 50 data sets according to maximum likelihood log-normal distributions created for each analyte using the bbmle and fitdistrplus packages. Hormone concentrations were analyzed with a linear mixed-effects model using lmer from the lme4 package and a glht *post hoc* test from the multcomp package. Treatment outcome and time point were fixed effects and patient identification a random effect. The assumption of the mixed-effect model was that the residuals after fitting the model were normally distributed. qqplots indicated outliers were present in estradiol, ghrelin, leptin, and DHEA with extreme outliers present in progesterone. To test the sensitivity of the models, outliers were removed using Cook’s distance metric until the Shapiro–Francia test, from the nortest package, did not detect deviation from the normal distribution at *p* ≤ 0.05. Results remained unchanged, i.e., statistical differences remained, after excluding the outliers. This was, however, not true for progesterone and we excluded the hormone from the analysis. Data are presented as means and SDs of the log-transformed data. To determine the linear relationship between different hormones and between hormones and cytokines, a Pearson correlation on quantile normalized data was used. Average Pearson correlation coefficients and *p*-values of 50 imputed data sets are reported. A principal component analysis (PCA) was used to evaluate the strengths of the relations between variables in cured and failed patients using the prcomp function and the bi-plots reported. Prediction models were developed using Elastic-Net regression analysis (with a parameter setting of alpha = 0.5) from the glmnet package. Variables that predicted hormone or cytokine concentrations in more than 60% of the 50 imputed datasets were included in the model.

Qlucore Omics Explorer (Lund, Sweden) was used to generate heat maps of the log-transformed means of the hormones and Ingenuity Pathway Analysis (IPA) (Qiagen, Redwood City, CA, USA) used to determine the canonical pathways in which inflammatory markers were involved.

## Results

### Baseline Characteristics

The aim of this study was to investigate immune and endocrine responses during the course of TB chemotherapy in patients with successful and unsuccessful treatment outcomes. BMI (kg/m^2^) was determined at each clinic visit and was previously shown to be a good predictor of poor treatment outcome (19). We found that the BMI increased, in both groups, during TB treatment, and were not different between the two groups at any of the three time points (Table 1). Corrections for BMI in subsequent analysis were therefore omitted. In addition, there were no differences with respect to age, sex distribution, and CXR score (Table 1).

**Table 1:** Characteristics of study groups.

	Cured (*n* = 27)	Failed (*n* = 10)	*p*-Value
BMI (kg/m^2^), BL	18.1 ± 2.1	18.8 ± 1.9	0.33
BMI (kg/m^2^), W4	18.5 ± 2.4	19.3 ± 1.5^b^	0.18
BMI (kg/m^2^), M6	19.5 ± 2.1^a^	19.8 ± 1.7^a^	0.68
Age (years)	35.0 ± 10.42	38.6 ± 11.9	0.44
Sex (F/M)	14/13	5/5	
CXR score	57.6 ± 31.3	71.7 ± 34.1	0.31

*Differences between groups were determined by a Mann–Whitney U test. Results are shown as mean ± SD*.

*BL, baseline; W4, week 4; M6, month 6; F, female; M, male; BMI, body mass index; CXR, chest X-ray*.

*^a^ and ^b^, significantly different from BL of the same group*.

### Hormone Profiles in TB Patients Changed during the Course of Treatment

**Figure 1:**
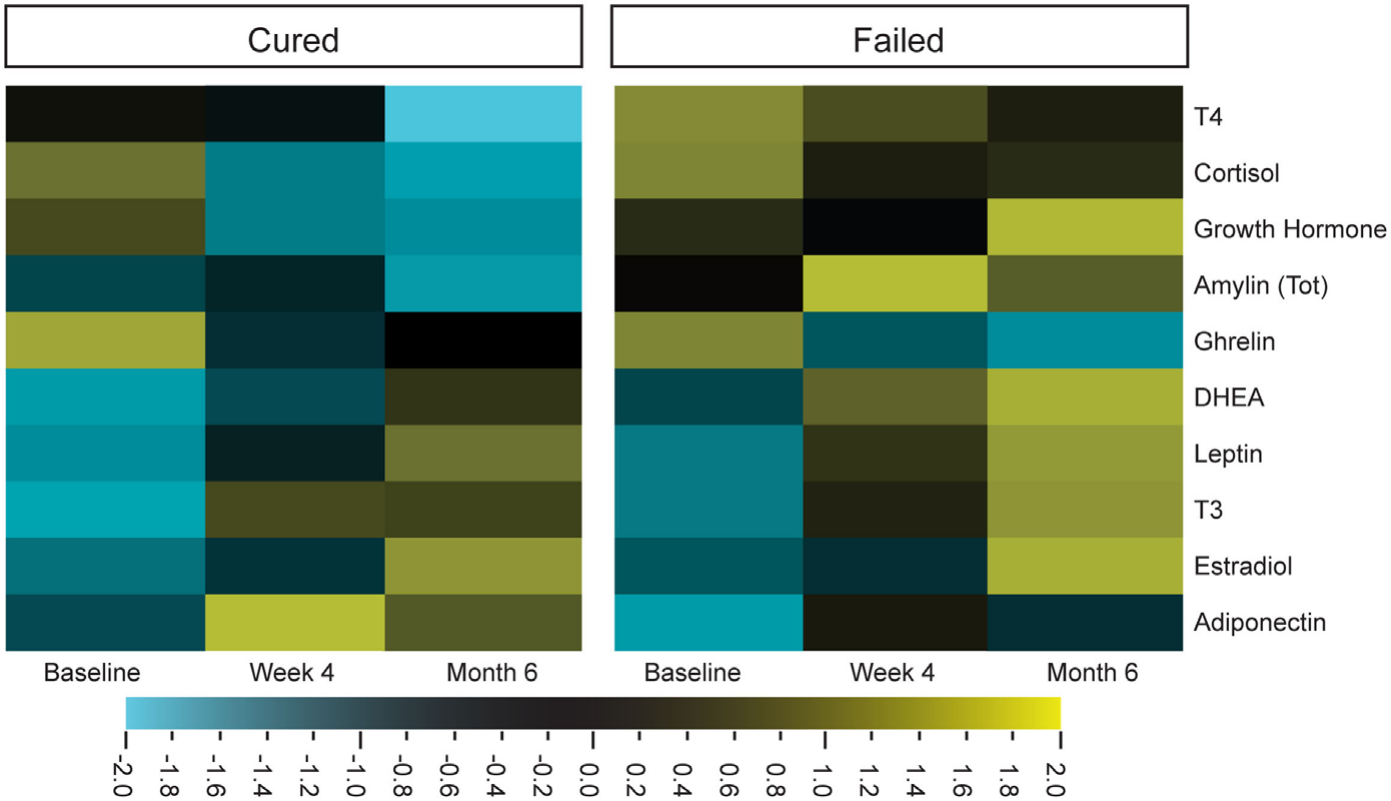
Differential expression of plasma hormone concentrations before (baseline), during (week 4), and after tuberculosis treatment (month 6). Hormone concentrations, measured by Luminex (cortisol, T3, T4, ghrelin, leptin, and estradiol) and ELISA [dehydroepiandrosterone (DHEA), amylin (Tot), adiponectin, and growth hormone], were clustered according to group and time point (cured group *n* = 25 and failed group *n* = 10). Qlucore Omics explorer software was used to generate the heat maps around the mean hormone concentrations of the log-transformed data. Bright yellow indicates +2 (fourfold) upregulation from the mean (black) and bright blue indicates a −2 downregulation from the mean.

Cortisol, DHEA, and leptin concentrations in particular were characterized in the context of TB, by comparing the hormone concentrations of TB patients to those of healthy controls (4, 10). Limited information is available on other endogenous hormones, especially on how their concentrations are affected by TB treatment. We thus investigated hormonal changes in individuals diagnosed with TB, during TB treatment. The hormone profiles of the two different treatment outcome groups were different from each other and different treatment responses were observed for cortisol, T4, amylin (total), and DHEA (Figure 1).

**Figure 2:**
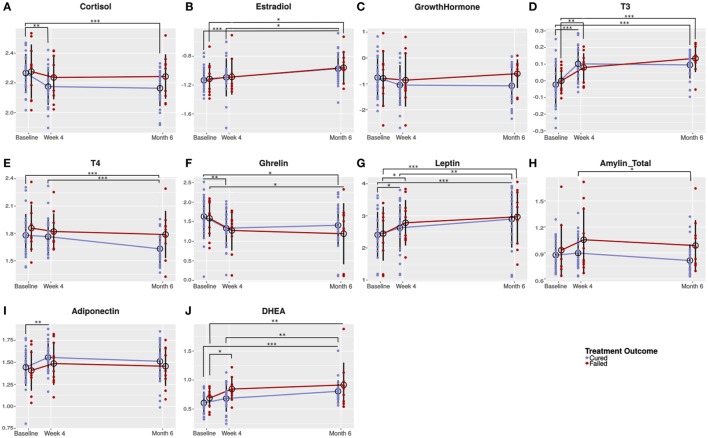
Changes in circulating hormone concentrations during the course of tuberculosis (TB) treatment. Plasma from TB patients (males and females) who were cured (*n* = 25) and who failed (*n* = 10) TB treatment were collected before (baseline) and during TB treatment (week 4 and month 6). Plasma collected was subjected to hormone measurement by Luminex analysis [cortisol **(A)**, T3 **(D)**, T4 **(E)**, ghrelin **(F)**, leptin **(G)**, and estradiol **(B)**] and ELISA [dehydroepiandrosterone (DHEA) **(J)**, amylin (Tot) **(H)**, adiponectin **(I)**, and growth hormone **(C)**]. Data were analyzed by a linear mixed-effects model of hormone concentrations by treatment outcome and time point as fixed effects and patient ID as random effect. Results are presented as means of the log-transformed data with SD. A p-value of < 0.05 was regarded as significantly different. *p-value < 0.05; **p-value < 0.01; ***p-value < 0.001.

Univariate analysis showed that cortisol concentrations significantly decreased in cured patients from baseline (before TB treatment) to week 4 and remained low until the end of treatment, while cortisol concentrations remained unchanged in the failed group (Figure 2A). The concentrations of the sex hormone estradiol, increased during TB treatment in both groups (Figure 2B). Since we observed the same treatment response in both genders, we reported the pooled data. No differences were detected in growth hormone concentrations (Figure 2C). T3 concentrations significantly increased in the failed group from baseline to week 4. The increase in the same groups from week 4 to month 6 did not reach statistical significance (Figure 2D). In the cured group, on the other hand, there was a significant increase only from baseline to week 4. TB treatment did not alter T4 concentrations in patients who failed treatment; however, in cured patients, there was a trend that T4 concentrations decreased resulting in month 6 concentrations being lower than in patients who failed treatment (*p* = 0.050; Figure 2E).

Ghrelin concentrations showed an early treatment response with a significant decrease from baseline to week 4 in the cured groups and a trend toward a decrease in the failed group. Thereafter, in both treatment groups, ghrelin concentrations remained unchanged (Figure 2F). In the cured group, leptin concentrations significantly increased from baseline to week 4 and again from week 4 to month 6 (Figure 2G). In the failed group, leptin concentrations significantly increased from baseline to week 4, but no difference was observed between week 4 and month 6. The increase in leptin observed during treatment is possibly due to the weight gain associated with TB treatment. There was a trend that amylin (total) concentrations increased from baseline to week 4 in the failed group and remained the same until the end of treatment (Figure 2H). In the cured group, amylin (total) concentrations remained unchanged from baseline to week 4 and then significantly decreased from week 4 to month 6. At 4 weeks (*p* = 0.083) and 6 months (*p* = 0.050), there were trends that amylin (total) concentrations were lower in cured patients when compared to the failed patient group.

Statistical significant changes in adiponectin concentrations were only observed in the cured patient group (Figure 2I). DHEA concentrations steadily increased during treatment in the cured group, whereas, in the failed group, there was a significant increase from baseline to week 4 (Figure 2J). At week 4, there was a trend that the failed group had higher DHEA concentrations than the cured group (*p* = 0.056).

**Figure 3:**
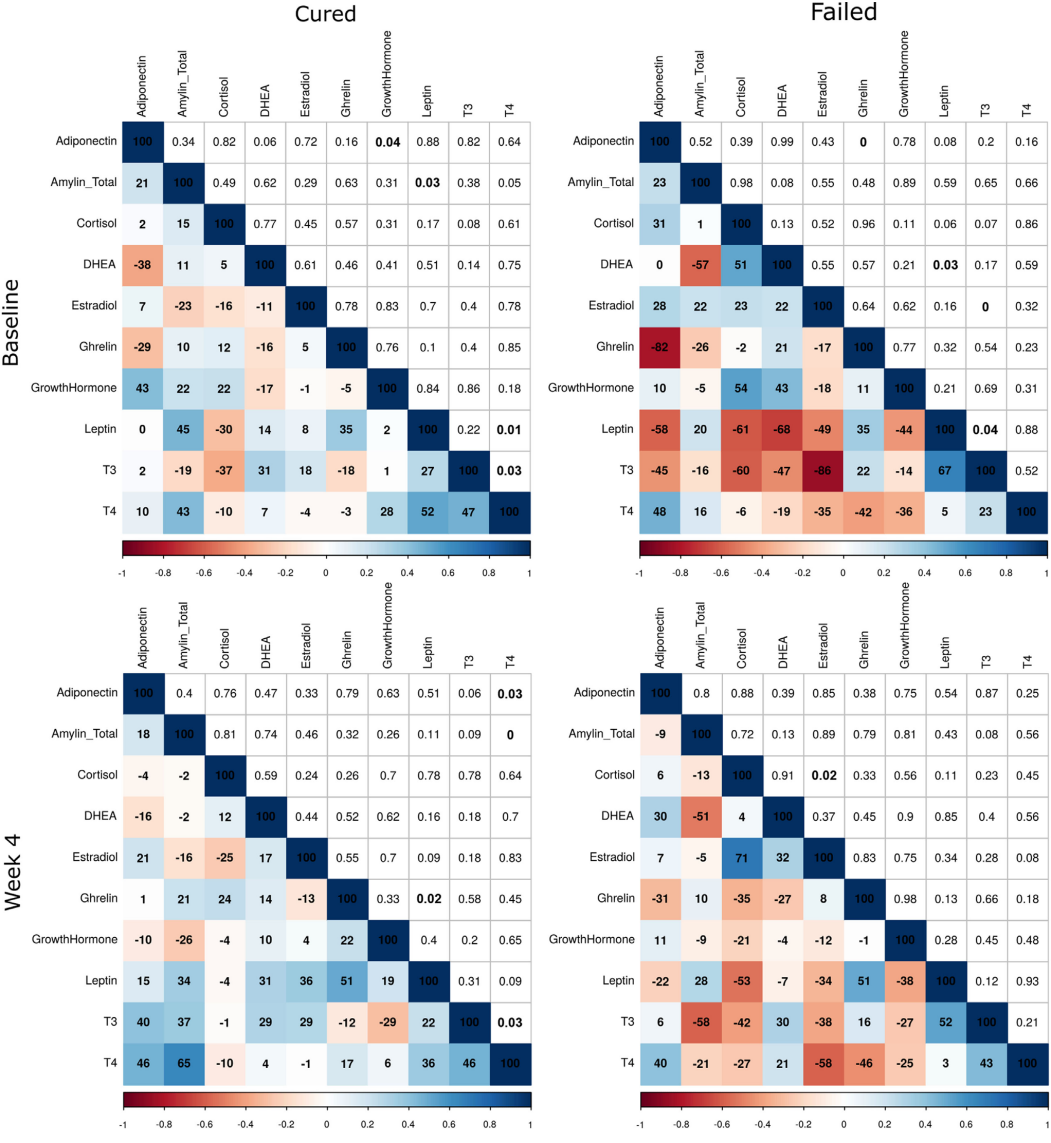

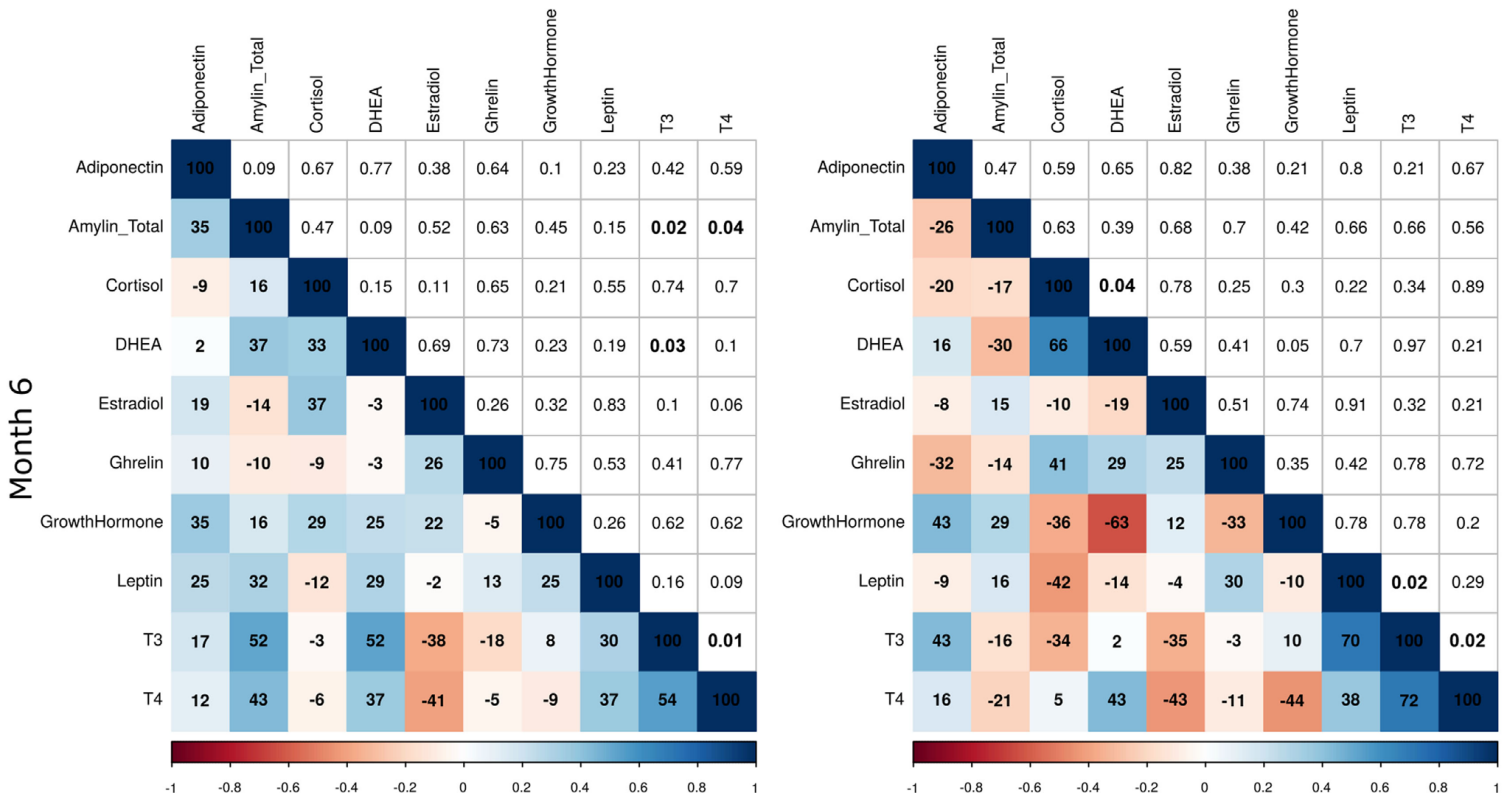
Correlations between plasma hormone concentrations of tuberculosis patients who were cured and who failed treatment at baseline, week 4, and month 6. Hormone concentrations were measured by Luminex analysis (cortisol, T3, T4, ghrelin, leptin, and estradiol) and ELISA [dehydroepiandrosterone (DHEA), amylin (Tot), adiponectin, and growth hormone] (cured: *n* = 25; failed: *n* = 10). Average Pearson correlation coefficients and *p*-values of 50 imputed datasets are presented. Correlation coefficients are expressed as percentages in the colored squares (bottom-left section) and the *p*-values in the white squares (top-right section). The colorimetric scale represents the correlation coefficient where dark blue squares indicate strong positive correlations (+1) and dark red strong negative correlations (−1).

Further differences were observed between the two treatment outcome groups when the correlation matrices of the hormones were compared at the three time points (Figure 3). The matrices were used to present the Pearson correlation coefficients and *p*-values. Changes in the intensity of the colors, representing the direction and significance of the correlations, indicated that there were differences in the hormone–hormone interactions between the two patient groups. As expected, differences were observed at baseline and week 4, but surprisingly there were still differences in the hormone interactions between the two groups at the end of treatment. With the major differences being the significant correlations in the failed group at baseline between ghrelin and adiponectin (*r* = −0.85, *p* = 0.00), leptin and DHEA (*r* = −0.68, *p* = 0.03), T3 and estradiol (*r* = −0.86, *p* = 0.00), and T3 and leptin (*r* = 0.67, *p* = 0.04). At month 6, the significant correlations in the failed group were DHEA and cortisol (*r* = 0.66, *p* = 0.04), growth hormone and DHEA (*r* = −0.63, *p* = 0.05), T3 with leptin (*r* = 0.70, *p* = 0.02), and T4 with T3 (*r* = 0.72, *p* = 0.02).

### During TB Treatment, Altered Hormone Concentrations Correlated with Immune Marker Concentrations

**Figure 4:**
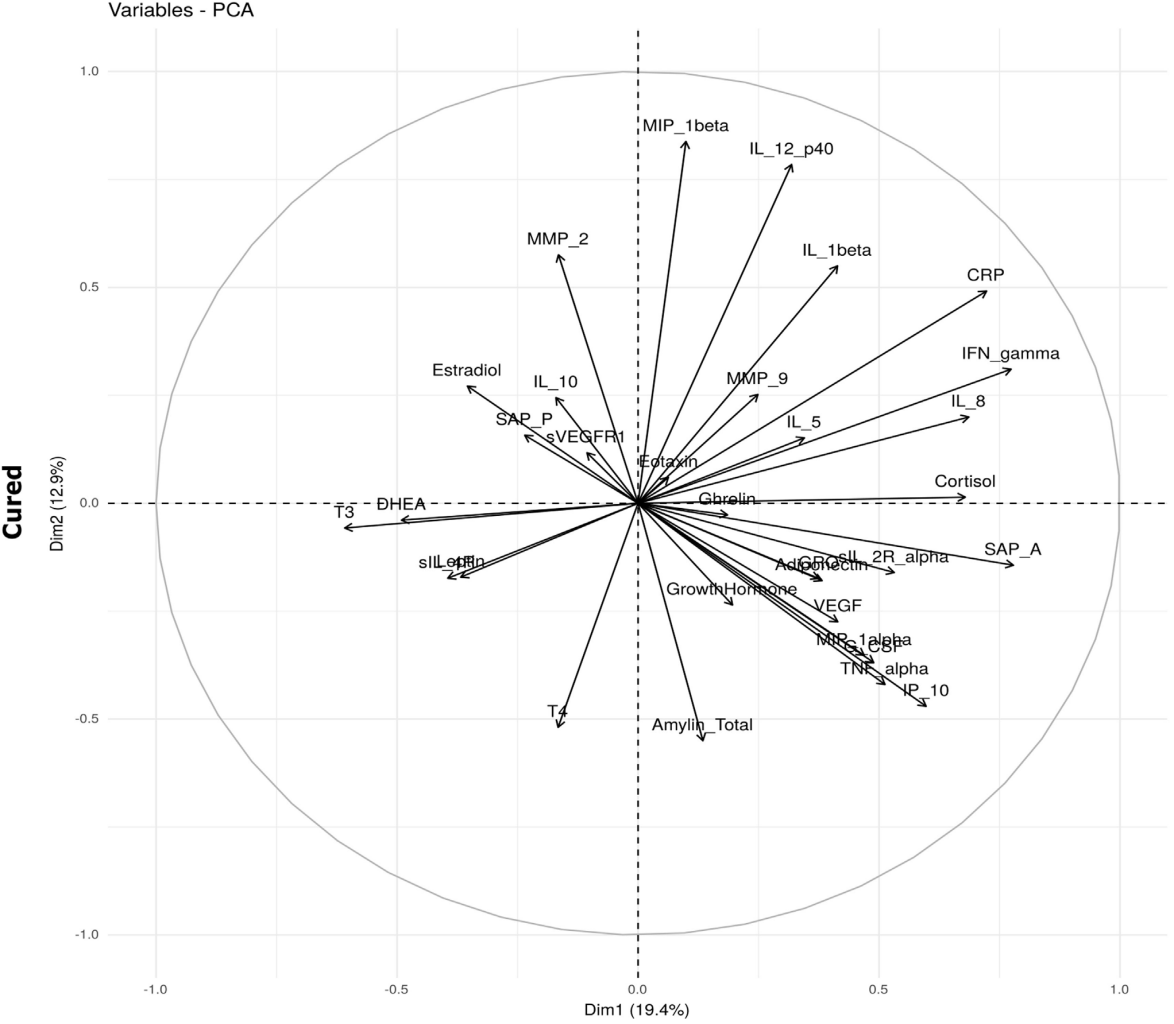

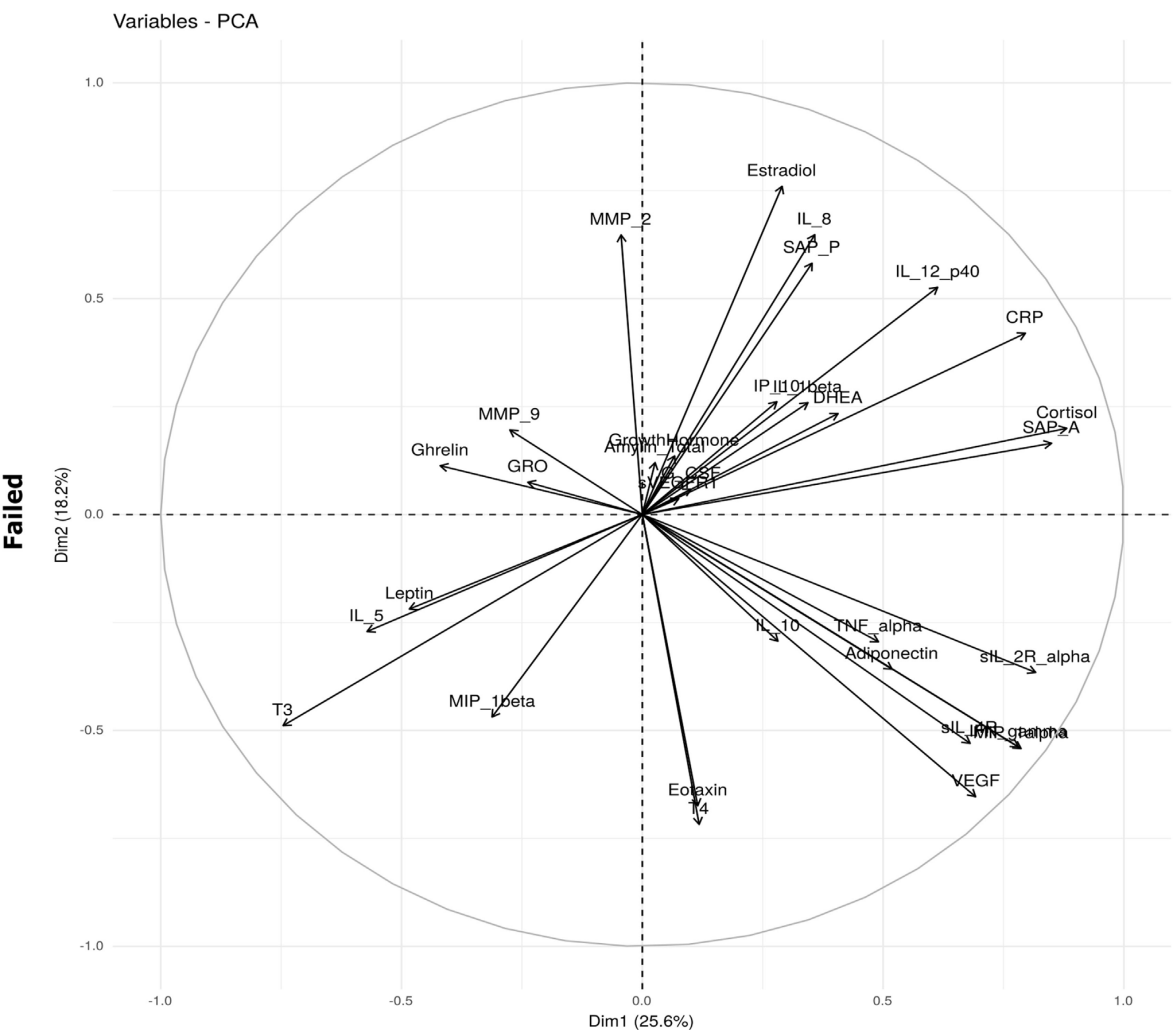
Bi-plots depicting plasma hormone and serum inflammatory marker interaction. Interactions between hormones and inflammatory markers were determined before tuberculosis treatment (baseline) in patients who were cured (*n* = 25) and those who failed (*n* = 10) treatment. The principal component analysis (PCA) was done using the prcomp function in R and the bi-plot generated using the factoextra package.

In an attempt to understand the immune–endocrine interaction during TB treatment, we investigated whether changes observed in hormone concentrations correlated with concentrations of different immune markers measured at each of the time points. We aimed to determine whether the interactions between immune markers and hormones were different in the two treatment outcome groups. Bi-plots from the PCA suggest that the relationships were different. For example, in the cured group, SAP A, IL-8, IFNγ, and CRP positively correlated with cortisol (Figure 4). It is possible that the increase in inflammatory markers during TB disease results in the increased production of cortisol by means of a feedback mechanism as increased cortisol concentrations subsequently prevent excessive immune responses and immunopathology. Cortisol furthermore negatively correlated with DHEA, T3, sIL-4R, and leptin. In the failed group, interactions between IFNγ, soluble receptors, DHEA, sex hormones, and cortisol varied from those observed in the cured group (Figure 4).

Unique combinations of inflammatory markers correlated with each of the hormones in the cured and failed treatment groups and again, differences between the groups were evident at all three time points (Tables 2–4). During TB treatment, the elevated cortisol concentrations in failed patients negatively correlated with MMP-9 at week 4, whereas in cured patients, a negative correlation was found at the end of treatment with IP-10. DHEA negatively correlated with inflammatory markers in the cured group at baseline and week 4. Interestingly, no correlations were found between DHEA and inflammatory markers in the failed group. Inflammatory markers negatively correlated with T3 in both the cured and failed groups at baseline. At week 4, inflammatory markers continued to negatively correlate with T3 in the cured group, but no correlations were found in the failed group. At the end of treatment, there were no correlations with T3 in cured group whereas, in the failed group, TNFα positively correlated with T3. T4 concentrations did not correlate with any inflammatory markers in the cured group, but positively correlated with markers at week 4 and month 6 in the failed group. High amylin (total) concentrations in the failed group positively correlated with sIL-2Rα at week 4, while concentrations of this hormone negatively correlated with MIP-1β at baseline in the cured group. These data point toward a dynamic interplay between the immune and endocrine system that is vastly different between the two treatment outcomes.

**Table 2:** Baseline correlations between hormones and inflammatory markers.

Hormone	Inflammatory marker (Pearson correlation coefficient and *p*-value)	
	Cured	Failed
Adiponectin	IP-10 (0.49; 0.01)	
Amylin (Tot)	*MIP-1β (−0.53; 0.02)*	
Cortisol	IL-8 (0.54; 0.02), TNFα (0.47; 0.03)	CRP (0.72; 0.02), SAP A (0.83; 0.00), sIL-2Rα (0.85; 0.00)
Dehydroepiandrosterone	*IL-5 (−0.47; 0.03), SAP A (−0.52; 0.01), CRP (−0.56; 0.00)*	
Estradiol	*VEGF (−0.49; 0.04)*	
T3	*IL-8 (−0.51; 0.03), sIL-2Rα (−0.51; 0.01)*	*CRP (−0.72; 0.02), IL-5 (0.65; 0.04)*

*Italic text: negative correlations*.

**Table 3:** Week 4 correlations between hormones and inflammatory markers.

Hormone	Inflammatory marker (Pearson correlation coefficient and *p*-value)	
	Cured	Failed
Adiponectin		IFNγ (0.93; 0.00), MIP-1α (0.75; 0.02), sIL-4R (0.74; 0.01), VEGF (0.75; 0.02), G-CSF (0.65; 0.04)
Amylin (Tot)		sIL-2Rα (0.67; 0.04)
Cortisol		*MMP-9 (−0.77; 0.01)*
Dehydroepiandrosterone	*CRP (−0.55; 0.00), IP-10 (−0.47; 0.02), SAP A (−0.54; 0.01), sIL-2Rα (−0.40; 0.04)*	
Estradiol	*IFNγ (−0.47; 0.04), IP-10 (−0.42; 0.04), SAP A (−0.46; 0.02), TNFα (−0.49;0.04)*	*GRO (−0.79;0.01), MIP-1β (−0.66; 0.04), IL-10 (−0.72;0.02)*
Ghrelin	GRO (0.54; 0.01)	*IP-10 (−0.74; 0.01)*, MMP-9 (0.65; 0.04)
T3	*CRP (−0.51; 0.02), sIL-2Rα (−0.56; 0.01)*	
T4		IP-10 (0.72; 0.02), MIP-1β (0.86; 0.00)

*Italic text: negative correlations*.

**Table 4:** Month 6 correlations between hormones and inflammatory markers.

Hormone	Inflammatory marker (Pearson correlation coefficient and *p*-value)	
	Cured	Failed
Adiponectin		IP-10 (0.83; 0.01)
Cortisol	*IP-10 (−0.47; 0.02)*	
Ghrelin	*MMP-2 (−0.43; 0.05), sIL-2Rα (−0.48; 0.02)*	
Growth hormone		IL-5 (0.72; 0.02)
T3		TNFα (0.69; 0.03)
T4		TNFα (0.65; 0.05)

*Italic text: negative correlations*.

The IPA software was used to identify the biological functions and immunological pathways in which the group of markers which correlated with a particular hormone, in a particular response group, at a particular time point were involved, regardless of whether there was a positive or negative correlation. Canonical pathways associated with the hormones in a group over all time points were pooled and the unique pathways, which distinguished the two patient groups, reported in Table 5. Pathways associated with hormones in the cured group were the inflammasome pathway, cytokine-mediated communication between immune cells, VEGF ligand–receptor interactions, and signaling of the myeloid cell receptor, triggering receptor expressed on myeloid cells 1 (TREM1). Pathways associated with hormones in the failed group were the adhesion and diapedesis of agranulocytes and granulocytes, hepatic fibrosis/stellate cell activation, and liver X receptor (LXR) and farnesoid X receptor (FXR) activation (Table 5). The inflammasome pathway in the cured group was associated with T3 concentrations at baseline that correlated with IL-8 and sIL-2Rα (Table 2). The role of cytokines in mediating communication between immune cells and the TREM1 signaling pathway were both associated with cortisol concentrations at baseline that correlated with IL-8 and TNFα. The VEGF family ligand–receptor interaction pathway was associated with estradiol concentrations that correlated with VEGF at baseline. In the failed group, agranulocyte adhesion and diapedesis were associated with ghrelin concentrations (week 4) correlating with IP-10 and MMP-9 (Table 3). Granulocyte adhesion and diapedesis was associated with adiponectin (week 4) that correlated with IFNγ, MIP-1α, sIL-4R, VEGF, and G-CSF, estradiol (week 4) that correlated with GRO, MIP-1β, and IL-10, and ghrelin (week 4) that correlated with IP-10 and MMP-9. Hepatic fibrosis/hepatic stellate cell activation was also associated with adiponectin concentrations at week 4, whereas the LXR/retinoid-X-receptor (RXR) and FXR/RXR activation pathway were associated with cortisol at baseline correlating with CRP, SAP A, and sIL-2Rα. This indicates that markers associated with cortisol, T3, and estradiol at baseline drive the unique pathways, identified in the cured group whereas markers correlating with ghrelin, adiponectin, and estradiol at week 4 and cortisol at baseline primarily drive the pathways unique to the failed group.

**Table 5:** Unique canonical pathways associated with inflammatory markers which correlate with the hormones in patients who were cured and patients who were not successfully cured after tuberculosis treatment.

Canonical pathways	
Cured	Failed
Role of cytokines in mediating communication between immune cells	Liver X receptor/retinoid-X-receptor (RXR) activation
VEGF family ligand–receptor interactions	Farnesoid X receptor/RXR activation
Inflammasome pathway	Granulocyte adhesion and diapedesis
Triggering receptor expressed on myeloid cells 1 signaling	Agranulocyte adhesion and diapedesis
	Hepatic fibrosis/hepatic stellate cell activation

*Canonical pathways associated with the hormones in a group over all time points were pooled and the unique pathways, which distinguished the two patient groups, listed here*.

### Hormones Predicted Cytokine Concentrations

In a further attempt to understand the interplay between the immune and endocrine system, we determined whether hormone concentrations could predict cytokine concentrations and *vice versa*. An Elastic-Net regression was used to determine which markers predicted which in 50 imputed datasets and predictions which occurred in at least 60% of the imputed datasets were reported. Not only did inflammatory markers predict hormone concentrations (Table 6) but some hormones also predicted inflammatory marker concentrations (Table 7). GRO and SAP A predicted ghrelin concentrations in 98% of the datasets, SAP P predicted ghrelin concentrations in 96%, and IL-12p40 in 92% of the datasets. SAP P predicted estradiol concentrations along with MMP-2 in 94 and 82% of the datasets, respectively. MMP-2 furthermore predicted adiponectin concentrations in 90% of the datasets. G-CSF predicted T4 and leptin concentrations in 94% of the datasets. As expected, TNFα and IFNγ predicted cortisol concentrations, and this was true in 80% of the imputed datasets. VEGF (60%) and its receptor, sVEGFR1 (88%), predicted IFNγ and amylin (total) concentrations.

**Table 6:** Cytokine predictors of hormone concentrations.

Hormone	Cytokines predicting hormone concentrations (%)
Cortisol	TNFα (0.80), IFNγ (0.80), IL-8 (0.70), sIL-2Rα (0.68), and VEGF (0.60)
Estradiol	SAP P (0.94), MMP-2 (0.82), and IL-10 (0.78)
T3	sIL-4R (0.70) and MIP-1β (0.68)
T4	G-CSF (0.94)
Ghrelin	GRO (0.98), SAP A (0.98), SAP P (0.96), and IL-12p40 (0.92)
Leptin	G-CSF (0.94), GRO (0.94), IL-10 (0.90), and IL-5 (0.70)
Amylin (Tot)	sVEGFR1 (0.88), IL-1β (0.78), VEGF (0.76), SAP A (0.72), and G-CSF (0.68)
Adiponectin	MMP-2 (0.90), IP-10 (0.82), IFNγ (0.82), and IL-12p40 (0.72)

*%: percentage of 50 imputed datasets the inflammatory marker predicted the concentrations of the corresponding hormone*.

**Table 7:** Hormone predictors of cytokine concentrations.

Cytokines	Hormones predicting cytokine concentrations (%)
G-CSF	T4 (0.96), cortisol (0.90), and ghrelin (0.82)
IL-12p40	Adiponectin (1.00) and ghrelin (0.98)
IP-10	Adiponectin (1.00), cortisol (1.00), ghrelin (1.00), T4 (1.00), and leptin (0.78)
TNFα	Cortisol (1.00) and T4 (1.00)
VEGF	Amylin (Tot) (0.78)
CRP	Cortisol (1.00), ghrelin (1.00), growth hormone (1.00), and T4 (1.00)
SAP P	Ghrelin (0.90)
IL-8	Cortisol (0.76)
IFNγ	Adiponectin (1.00), cortisol (1.00), T4 (0.98), and ghrelin (0.66)
sIL-2Rα	Cortisol (1.00) and T4 (1.00)
sVEGFR1	Amylin (Tot) (0.76)
MMP-2	Adiponectin (1.00), estradiol (1.00), T3 (1.00), and dehydroepiandrosterone (0.94)

*%: percentage of 50 imputed datasets the hormone predicted the concentrations of the corresponding inflammatory marker*.

Hormones were also good predictors of inflammatory marker concentrations. Adiponectin predicted IL-12p40 concentrations in all the datasets. Adiponectin, cortisol, ghrelin, and T4 predicted IP-10 concentrations; cortisol and T4 predicted TNFα concentrations, cortisol, ghrelin, growth hormone, and T4 predicted CRP concentrations; adiponectin and cortisol predicted IFNγ concentrations; cortisol and T4 sIL-2Rα concentrations and adiponectin, estradiol, and T3 predicted MMP-2 concentrations, all with the same frequency.

## Discussion

By exploring a wide range of biological markers, including hormones and inflammatory molecules, a better understanding of pathogen-induced immunopathology during TB can be attained. In an attempt to understand these interactions during *M.tb* infection, Santucci et al. investigated the multifaceted immune–endocrine–metabolic alterations in patients with different severities of pulmonary TB (11). Bongiovanni et al. reported the immune–endocrine responses during TB treatment (15). However, both these studies investigated a limited number of hormones and immunological markers.

In this report, we included a range of hormones, immunological markers, and comprehensive correlations to investigate the immune–endocrine interaction. We found that the hormone profile of individuals who are successfully cured after completing the 6-month TB treatment regimen differed from those who failed TB treatment. We showed that estradiol, T3, leptin, and DHEA concentrations increased and that cortisol, T4, ghrelin, and amylin (total) concentrations decreased during TB treatment. Cortisol, T4, amylin (total), and DHEA concentrations remained lower in the cured patients. We furthermore showed that hormone concentrations correlated with different groups of immunological markers. We were able to identify immunological pathways for the groups of markers that correlated with hormones in the different patient groups. This study, however, has several limitations such as the small sample sizes and the fact that dietary intake and blood collection time was not standardized. Not only hormone but also cytokine concentrations, for instance, IL-1β are known to change post-prandially (20), yet this is almost never considered in TB biomarker studies available in the literature and should be taken into account in any future studies. Despite the limitations, typical treatment responses were observed when looking at the changes in hormone concentrations during TB treatment and it would appear that cortisol, DHEA, T4, and amylin (total) are the major role players in the host response to *M.tb* and are potential marker candidates to differentiate treatment outcome.

Cortisol concentrations are higher in TB cases than in healthy controls at baseline and increase as disease severity increases (4, 11). DHEA concentrations on the other hand are lower in TB cases than controls and further decrease as disease severity increases (4). In keeping with and extending these results, we found that cortisol concentrations decreased during TB treatment in cured patients, but remain unchanged in patients with a failed treatment outcome, whereas DHEA concentrations suddenly increased in the failed treatment outcome group from baseline to week 4 and gradually increase in the cured group during treatment. We showed that cortisol concentrations positively correlated with acute phase proteins and soluble cytokine receptors before TB treatment. Cytokines produced during the course of the immune response induce alterations in the concentrations of hormones produced by the HPA, HPT, and HPG axis (21). The positive correlation observed between cortisol and inflammatory markers at baseline forms part of a feedback mechanism to limit the inflammatory response associated with active disease.

T3 and its precursor, T4, are primarily involved in metabolism and T3 promotes the production of pro-inflammatory markers (22). T3 and T4 also promote the catabolic state in TB patients (4). In some studies, T3 and T4 concentrations were found to be higher in TB patients (4, 23), while in others they were lower in TB patients and increased during TB treatment (24, 25). Extremely low T3 concentrations have been further associated with mortality in TB patients (25). We found that T3 concentrations increased during TB and that T4 concentrations remained unchanged in the failed group. T4 concentrations were lower in the cured group, compared to the failed group, at month 6.

Leptin concentrations are lower in TB patients (11) and low leptin concentrations and increased IL-6 concentrations are associated with wasting and weight loss in TB patients (26). Santucci et al. found a positive correlation between leptin concentrations and BMI (11), while another study found no correlation between these two parameters (27). Animal experiments suggest that leptin contributes to protection against *M.tb* by inducing Th1 cytokine responses (28). Such a role has not yet been conclusively shown in humans (11, 26, 29, 30). Some have suggested that leptin does not form part of the pro-inflammatory response (30) and in agreement with this, leptin did not correlate with any of the inflammatory markers investigated in this study. Ghrelin, which is produced by the stomach and acts as an orexigenic factor (31), is present at higher concentrations in TB patients and the increase in ghrelin concentrations could act as a compensatory mechanism to weight loss (27). Ghrelin can furthermore downregulate pro-inflammatory cytokine responses (32, 33). Our results suggest that ghrelin is involved in cytokine responses during TB treatment as some markers correlated with ghrelin.

Amylin, which is secreted in conjunction with insulin to maintain blood glucose concentrations, has been found to be higher in obese individuals than in their normal-weight counterparts and was positively associated with BMI and inflammatory markers like CRP and IL-6 (34). Our study, the first to measure amylin in TB patients, showed that amylin concentrations were higher in the failed group, during treatment, than in the cured group. Amylin concentrations were not only associated with inflammatory markers but have been found to induce the production of acute phase proteins (35) and to activate the NLRP3 inflammasome (36). We found amylin to be positively and negatively correlated with inflammatory markers in the failed and cured group, respectively, indicating that this hormone plays an important role in immunity to TB.

Distinct biological pathways were associated with the two treatment outcome groups. Cytokines in the cured group were associated with components of the innate immune system like the inflammasome pathway and TREM1 receptor. Gain-of-function gene variants in components of the inflammasome like the *NLRP3* gene and CARD8 gene enhance inflammasome activity and limit *M.tb* growth in human macrophages (37). In addition, the adaptor protein PYCARD (ASC) plays an important role in granuloma formation (38), while *M.tb* prevents inflammasome activation and IL-1β production to survive in macrophages (39). There was furthermore an association with VEGF signaling in cured patients. It has been shown that the expression of genes involved in angiogenesis is upregulated and the expression of VEGF-A, a key regulator of angiogenesis, has been found to be higher in *M.tb-*infected macrophages which promoted the formation of blood vessels (40). Inhibition of VEGF reduces dissemination of *M.tb*, and it is believed that mycobacteria exploit macrophages to disseminate by inducing the formation of blood vessels. VEGF has also been observed to be higher in patients with pulmonary TB, and it has been classified as a biomarker to distinguish latent infection and active disease (40, 41). It is therefore possible that the favorable treatment outcome in cured patients is due to early inflammasome involvement and the inhibition of the angiogenesis pathway.

Liver X receptors activation, associated with failed group, is known to regulate lipid metabolism and antimicrobial responses. DNA sequencing of the LXRA and LXRB receptors revealed common variants of which some were potential risk haplotypes while others were protective for TB (42). LXRs therefore play in important role in the genetic susceptibility to TB and possibly treatment outcome.

Changes observed in the cured group, such as the decrease in cortisol level and the gradual increase in DHEA, together with the markers associated with these hormones in this group, were possibly indicative of the restoration of the immunological responses during TB treatment and translated to the improvement of the clinical conditions of the patients. These results, furthermore, suggested that the inflammatory markers and the hormones were differentially regulating each other.

This study showed that hormone concentrations changed as a result of active TB and continued to change during TB treatment and that hormone profiles of individuals who were successfully cured differ from those who failed treatment. This study highlights the intricate relationships between the endocrine and immune systems during TB and support the suggestion that hormones in conjunction with cytokines may be valuable as biomarkers for treatment response.

## Ethics Statement

Ethical approval was obtained from the Health Research Ethics Committee of the University of Stellenbosch and the City of Cape Town City Health. The study was conducted according to the Helsinki Declaration and International Conference of Harmonization guidelines. Written informed consent was obtained from all study participants.

## Author Contributions

Conceived and designed the experiments: KR, LK, and GW. Performed the experiments: SR, LK, LE, LT, NC, and MKL. Analyzed the data: GS and MKD. Contributed reagents/tools: PH, KR, LK, GS, and MKD. Wrote the paper: LK. Critical review of manuscript and intellectual contributions: KR, PH, GW, GS, and MC.

## Conflict of Interest Statement

The authors declare that the research was conducted in the absence of any commercial or financial relationships that could be construed as a potential conflict of interest. The reviewer, VC, and handling editor declared their shared affiliation, and the handling editor states that the process nevertheless met the standards of a fair and objective review.

## Abbreviations

BMI, body mass index; CRP, C-reactive protein; CXR, chest X-ray; DHEA, dehydroepiandrosterone; FXR, farnesoid X receptor; IL, interleukin; LXR, liver X receptor; MMP, matrix metalloproteinase; *M.tb, Mycobacterium tuberculosis*; PCA, principal component analysis; RXR, retinoid-X-receptor; TB, tuberculosis; TNF, tumor necrosis factor; TREM1, triggering receptor expressed on myeloid cells 1.
